# BugSeq: a highly accurate cloud platform for long-read metagenomic analyses

**DOI:** 10.1186/s12859-021-04089-5

**Published:** 2021-03-25

**Authors:** Jeremy Fan, Steven Huang, Samuel D. Chorlton

**Affiliations:** BugSeq Bioinformatics Inc, Vancouver, BC Canada

**Keywords:** Metagenomics, Nanopore, Microbiology, Sequencing, Third-generation, Long-read

## Abstract

**Background:**

As the use of nanopore sequencing for metagenomic analysis increases, tools capable of performing long-read taxonomic classification (ie. determining the composition of a sample) in a fast and accurate manner are needed. Existing tools were either designed for short-read data (eg. Centrifuge), take days to analyse modern sequencer outputs (eg. MetaMaps) or suffer from suboptimal accuracy (eg. CDKAM). Additionally, all tools require command line expertise and do not scale in the cloud.

**Results:**

We present BugSeq, a novel, highly accurate metagenomic classifier for nanopore reads. We evaluate BugSeq on simulated data, mock microbial communities and real clinical samples. On the ZymoBIOMICS Even and Log communities, BugSeq (F1 = 0.95 at species level) offers better read classification than MetaMaps (F1 = 0.89–0.94) in a fraction of the time. BugSeq significantly improves on the accuracy of Centrifuge (F1 = 0.79–0.93) and CDKAM (F1 = 0.91–0.94) while offering competitive run times. When applied to 41 samples from patients with lower respiratory tract infections, BugSeq produces greater concordance with microbiological culture and qPCR compared with “What’s In My Pot” analysis.

**Conclusion:**

BugSeq is deployed to the cloud for easy and scalable long-read metagenomic analyses. BugSeq is freely available for non-commercial use at https://bugseq.com/free.

**Supplementary Information:**

The online version contains supplementary material available at 10.1186/s12859-021-04089-5.

## Background

Nanopore sequencing has seen a dramatic increase in read quality and throughput over the last few years, leading to increased adoption and novel applications. Recently, nanopore sequencing has been used for metagenomics in clinical, environmental and agricultural settings [1–4]. Many metagenomic read classifiers, originally designed for short (< 300 bp), high quality reads rely on k-mers for sequence classification [5]. Due to the high error rate of nanopore sequencing and the low likelihood of many consecutive error-free bases, k-mer methods are unlikely to be optimal for nanopore read classification [1]. Additionally, popular k-mer methods such as Kraken2 discard information on the order of k-mers within a sequence, which is useful data for classifying long reads [6].

Alternative methods have been explored: EPI2ME, a platform operated by Metrichor, uses Centrifuge as its classifier [7]. Centrifuge can start with short k-mer matches and extend them until the first nucleotide difference in alignment, enabling variable length matches. By default, Centrifuge starts this extension with 22 bp seeds, however this parameter can be set down to 16 bp for increased sensitivity. MetaMaps relies on approximate read alignment with minimizers and a probabilistic model to estimate sample composition [8]. Finally, CDKAM, a recently released tool, uses inexact k-mer matches to identify matches in a reference database [9].

These tools have previously been evaluated in benchmarks, and while they can provide taxonomic classifications, they each suffer from limitations [10]. Centrifuge and CDKAM are unable to provide accurate species-level classification, and report a large number of false positive read classifications [9, 10]. MetaMaps can provide more accurate classifications but suffers from long processing times (over 10 h for just 74,000 reads in a recent benchmark) [8]. All of these tools require large servers with more RAM than high-end consumer-grade computers [10]. These limitations are barriers to the widespread adoption of long read technology for metagenomic sequencing. For instance, even the identification of one read belonging to a pathogen can be significant in food, military or clinical samples, and these results must be available rapidly to enable action. We aim to overcome these limitations with a cloud-based, rapid, highly accurate metagenomic classifier for long reads.

## Methods

### Implementation

**Fig. 1:**
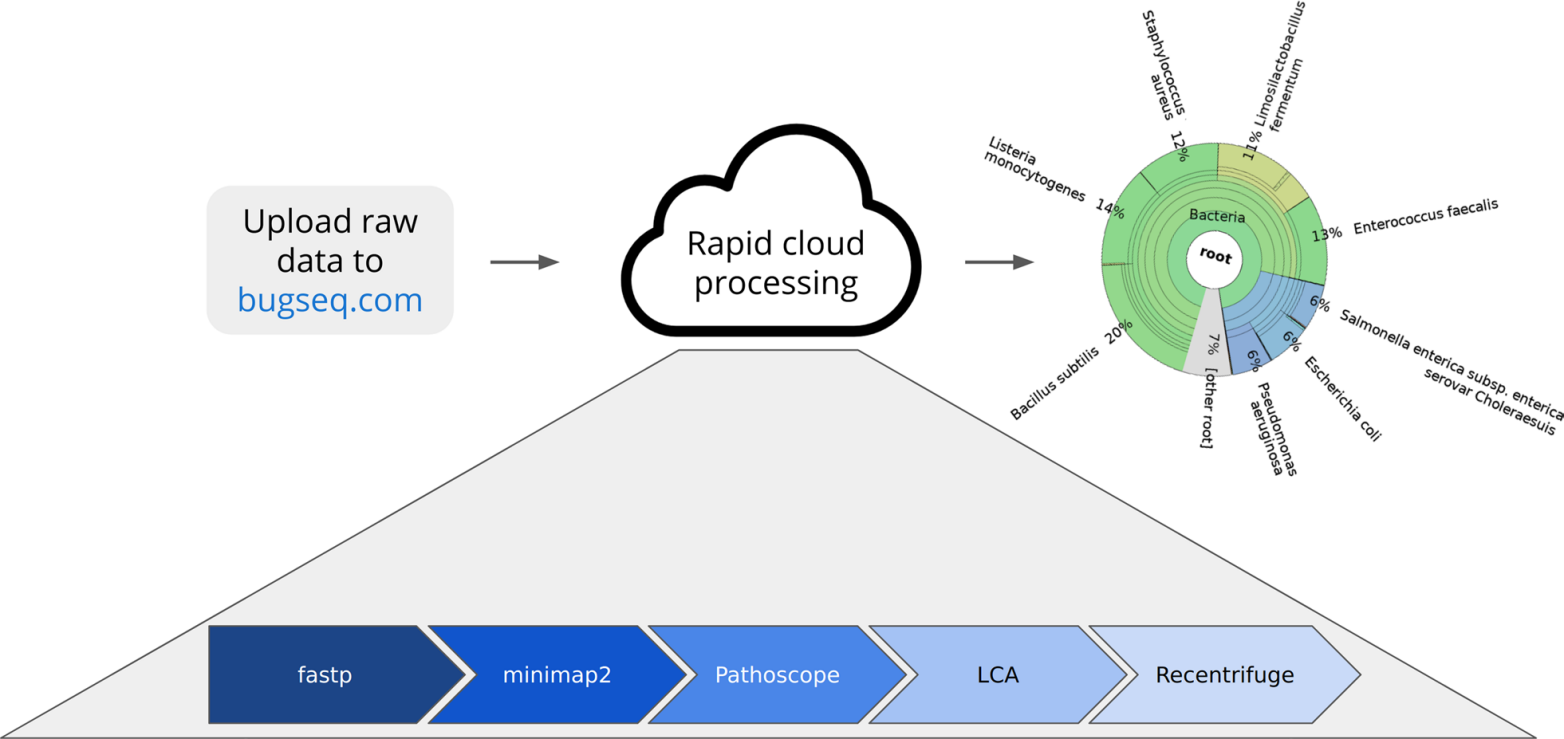
Overview of the BugSeq workflow

We combined a fast and accurate read mapper, Bayesian reassignment of reads based on mapping quality, a new lowest-common ancestor process, and an advanced visualization tool to build a better metagenomic classifier for nanopore reads. This pipeline, which we call BugSeq, has been packaged with Nextflow (v20.07.1) and made available as an online service (https://bugseq.com/free) for easy cloud analyses. An overview of the pipeline is presented in Fig. 1. In brief, reads are quality controlled with fastp (v0.20.1), using a minimum average read quality of Phred 7, a minimum read length of 100 bp, and the default low complexity filter [11]. Reads are then mapped with minimap2 (v2.17) to an index containing all microbes in RefSeq, the human genome, and a database of contaminants [12]. Specifically, this database contains all complete bacterial genomes; and all fungal, viral, protozoal and archaeal genomes found in RefSeq, regardless of completion status. Additionally, the human genome and a database of contaminants (Univec) are included. The database for evaluation was generated on February 23, 2020 but has since been updated monthly. For use with an alternative reference database, please get in touch with the corresponding author. Minimap2 was executed in “map-ont” mode with the “-a” flag to align reads into SAM format. A range of parameters was evaluated for minimap2, including varying the number of secondary alignments. Most variations of this parameter, from 5 to 25, produced little variation in the final results; 10 was selected based on a good trade-off between accuracy and speed, and was used for all subsequent analyses (data available at https://gitlab.com/bugseq/metagenomicclassifiersbenchmarking/-/tree/master/program_outputs/BugSeq/param_search). Next, alignments to the reference sequences are reassigned based on a Bayesian statistical framework using Pathoscope (version 2.0.7) and default parameters [13]. Finally, the lowest common ancestor of reassigned reads is calculated and inputted into Recentrifuge (v1.1.1), setting the minimum required taxa to 1, and generic input mode for summarization and visualization [14]. Quality control results are summarized with MultiQC using a custom configuration which changes the report title and Phred thresholds for bad quality data to 7 [15]. All dependencies are packaged in Docker images, and jobs are executed on Amazon Web Services Batch in a secure, private environment.

### Evaluation

We evaluate BugSeq and alternative long read metagenomic classifiers using simulated data, and sequencing data from mock microbial communities and real patient samples. We generate simulated data using the CAMISIM package and its included mini_config.ini configuration [16]. This simulator generates microbial communities using the included 24 genomes, sampling abundance using a lognormal distribution at default values (*μ* = 1 and *σ* = 2). The read simulator was switched to NanoSim to generate nanopore sequencing reads, and one sample with 100 Mb of data was generated [17]. The *E. coli* simulation profile included with NanoSim was used for read simulation. Resulting simulated data, along with ground truth classifications, is available at: https://doi.org/10.5281/zenodo.4382659

We run the metagenomic classifiers included in our evaluation with the following commands:

CDKAM version 1.1: ‘./CDKAM.sh DB INPUT_FILE OUTPUT_FILE ---fastq nthread 32’.

MetaMaps commit 98102e9e684efa6a9903d8abe93600132c101ad0: ‘metamaps mapDirectly -t 32 --all -r databases/miniSeq+H/DB.fa -q INPUT_FILE -o TEMP_FILE --maxmemory 196 && metamaps classify -t 32 --mappings TEMP_FILE --DB databases/miniSeq+H’. Maximum memory was set to 70% of true maximum memory as recommended by the MetaMaps authors.

Centrifuge version 1.0.4-beta: ‘centrifuge -t -k 1 -p 32 -x DB -U INPUT_FILE -report-file OUTPUT_REPORT -S OUTPUT’. We specify the ‘-k 1’ flag to collapse reads to their lowest common ancestor. Default databases and parameters were used for all tools unless otherwise specified. For default databases, we use the following:

Centrifuge: the “h+p+v+c” database (https://doi.org/10.5281/zenodo.3732127)

MetaMaps: the original authors’ miniSeq+H database (https://doi.org/10.17605/OSF.IO/XY4VN).

CDKAM: the standard database generated on December 15, 2020.

All tools were evaluated using 32 threads and 280 Gb of RAM (our server capacity). All classifier outputs are available at https://gitlab.com/bugseq/metagenomicclassifiersbenchmarking.

We use precision and recall to evaluate the classification accuracy of each tool. Precision (the positive predictive value) was defined by the number of correctly called reads divided by the total number of classified reads at the specified rank. Recall was defined as the number of correctly called reads divided by the total number of reads (unclassified and classified) at any rank. F1 score was calculated as 2 × (Precision × Recall)/(Precision + Recall). Recall, precision and F1-scores for the ZymoBIOMICS mock community evaluation were calculated by considering reads assigned to expected nodes as correct at that taxonomic rank and any rank above, otherwise the read was considered incorrect. A list of expected nodes is provided in supplementary Gitlab repo (https://gitlab.com/bugseq/metagenomicclassifiersbenchmarking). Processing time and memory were calculated using the linux time utility, with the “verbose” option. Analysis of BugSeq run time by input size was performed with an AWS Batch worker instance warmed up to ensure comparable times across all input sizes. Reads were randomly samples from the ZymoBIOMICS LOG dataset with seqkit using the command “seqkit sample -j 32 -2 -n NREADS -o NREADS_reads.fastq -s 11 ERR3152364.fastq.gz” [18].

To evaluate abundance estimation, we calculate the root mean squared error (RMSE) as the square root of the averaged squared residuals. The lower the RMSE, the closer the forecast is to representing the actual data. Exact calculation is available in the Additional file 6: Gitlab repository. Identified abundance of each expected organism was calculated as the proportion of reads assigned to that species divided by the total number of reads assigned to any species in the sample.

We compare these proportions to the ZymoBIOMICS “Genome Copy”, which adjusts expected abundance for genome length [19].

For the evaluation of classifier performance on lower-respiratory tract infections, we download data from SRA accession PRJEB30781 [20]. We ensure comparability with the original authors of the data by only considering respiratory pathogens (defined in their Methods section) [20]. The following pathogens were considered significant and included in analysis: “*E. aerogenes*, *E. cloacae* complex, *E. coli*, *H. influenzae*, *K. oxytoca*, *K. pneumoniae*, *M. catarrhalis*, *P. mirabilis*, *P. aeruginosa*, *S. marcescens*, *S. aureus*, *S. pneumoniae* and *S. pyogenes*”. Sensitivity was calculated as the number of respiratory pathogens detected divided by the expected number detected, summed across all samples. Specificity was calculated as the number of respiratory pathogens called as not present in each sample (maximum 13), divided by the expected number called as not present, summed across all samples. The Charalampous et al. specificity calculation method calculated the number of specimens called as normal respiratory flora (NRF) or no growth (NG), divided by the true number of NRF/NG specimens (n = 6).

## Results

We assessed the performance of BugSeq and compared it with three competing tools: Centrifuge, MetaMaps and CDKAM [7–9]. We first assessed the performance of BugSeq on simulated data with known ground truth classifications. We generate a realistic community and nanopore metagenomic sequencing of it using the recently released CAMISIM tool [16]. We evaluate each metagenomic classifier using their default database as specified in the Methods section. BugSeq outperforms all tools on this simulated data when examining the species-level F1-score. BugSeq obtains a species-level F1 of 0.964 with 100% precision and 93.1% recall. In contrast, Centrifuge obtained a species-level F1 of 0.962 (precision: 99.0%, recall: 93.5%), MetaMaps 0.952 (precision: 99.8%, recall: 91.1%) and CDKAM 0.938 (precision: 96.8%, recall: 91.0%).

We next evaluated BugSeq and alternative tools using real nanopore sequencing data from two microbial communities with known composition. The ZymoBIOMICS mock communities contain 8 bacteria and 2 yeasts in even (hereafter referred to as “Even”) and logarithmic (hereafter referred to as “Log”) concentrations [21]. Sequencing data for both samples was independently generated on a GridION using the R9.4.1 chemistry and is publicly available (https://github.com/LomanLab/mockcommunity#data-availability). Full details regarding data generation and characteristics are available from the original publication [21]. All evaluations on the ZymoBIOMICS datasets used a reference database from RefSeq, as previous studies have demonstrated impact of the choice of reference database on classification performance (see “Methods” section for database details) [22].

At the species level, BugSeq attained the top precision and recall compared with MetaMaps, CDKAM and Centrifuge across both Even and Log datasets (F1-score_Even_: 0.95, F1-score_Log_: 0.95) (Table 1). On average, BugSeq had 6% better recall than MetaMaps while maintaining superior precision, and 2–5% better precision than Centrifuge while maintaining superior recall. Additionally, BugSeq had an average 2% better F1 than CDKAM. When analyzing the number of unique species identified by each tool (true count = 10), BugSeq found a total of 117 and 52 species in the Zymo Even and Log dataset, respectively. In comparison, MetaMaps identified 2144 (Log) and 1386 (Even) unique species using the “miniSeq + H” database and exceeded our RAM threshold with the RefSeq database (Table 1). Centrifuge identified 5380 and 5513, and CDKAM identified 3721 and 2956 species in the Even and Log datasets, respectively. Full results at each taxonomic rank can be found in Additional file 1: Table S1.

**Table 1:** Performance of four metagenomic classifiers (BugSeq, MetaMaps, Centrifuge, and CDKAM) on GridION ZymoBIOMICS Mock Log and Even communities

	Dataset used	F1 score at species level	Precision at species level (%)	Recall at species level (%)	Number of unique species detected (true count = 10)	Run time (wall clock) (hh:mm)	Classification memory requirement (GB)	Indexing time (wall clock) (hh:mm)	Indexing memory requirement (GB)
BugSeq	Even	**0.95**	**99.82**	**90.36**	**117**	4:09	118	1:15	243
	Log	**0.95**	**100**	**90.88**	**52**	4:25	113		
MetaMaps (miniSeq + H)	Even	0.89	99.13	80.71	2144	86:20	172.6	**Prebuilt**	**Prebuilt**
	Log	0.94	99.46	88.25	1386	125:22	184.0		
MetaMaps (RefSeq)	Even	Out of memory	NA	NA					
	Log	0.94	99.73	88.66	2094	154:29	270		
Centrifuge – 16 bp minimum hit length, -k 1	Even	0.79	93.00	69.17	4337	00:19	67.7	19:45	168
	Log	0.92	95.93	89.07	5777	00:19	64.4		
Centrifuge – default (22 bp) minimum hit length, -k 1	Even	0.80	94.71	68.53	5380	00:16	56.9		
	Log	0.93	97.14	88.65	5513	00:14	55.7		
CDKAM (standard + human)	Even	0.91	96.52	86.53	3721	**00:09**	**34.5**	28:23	66
	Log	0.94	99.06	89.2	2956	**00:08**	**34.5**		

Different k-mer seed sizes were explored for Centrifuge (16 bp and 22 bp), and two different databases were examined for MetaMaps (miniSeq + H and RefSeq); otherwise, default parameters were used. The best result for each column and dataset combination is bolded

**Fig. 2:**
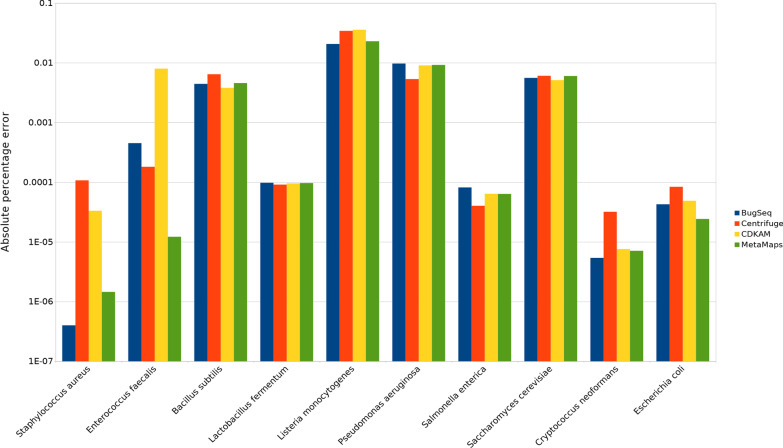
Absolute percent error of abundance estimate for each organism and tool when evaluated on the Zymo Log dataset

We next evaluated the ability of each tool to estimate taxonomic abundance. We use the Zymo Log dataset from above, as it contains organisms across a broad range (10^−2^ to 10^8^ cells) of abundance. For each organism in the sample, we compare the abundance calculated by the taxonomic classifier to the expected abundance of that organism. The absolute percentage error for each species and classifier combination, calculated as the difference between the expected and identified abundance, are presented in Fig. 2. We use the root mean square error to quantify the overall performance of each tool. Again, BugSeq performs better than alternative tools, with a RMSE of 0.0076, followed by MetaMaps (0.0082), Centrifuge (0.011) and CDKAM (0.012). Of note, Centrifuge demonstrates its weakness with long read data here; with its built-in abundance calculation, it assigns all taxa in the sample an abundance of 0, except for *Hungateiclostridium saccincola*, which had a total of 20 reads assigned (0.0006% of total) and is given an abundance of 1.

We next measured computational performance for all tools. BugSeq is an order of magnitude faster than MetaMaps, which took over 5 days using 32 cores and their “miniSeq + H” database. BugSeq took up to 4 h and 25 min to analyse the same amount of data. Notably, MetaMap’s miniSeq + H (26 GB) is significantly smaller than RefSeq (86 GB). BugSeq had longer run times than Centrifuge, which took 14 to 19 min, and CDKAM, which took 8 to 9 min, for all analyses. All tools required more than 32 GB of RAM for execution, precluding their use on modern laptops. An analysis of BugSeq’s run time by input size is provided in Additional file 1: Table S3.

To evaluate BugSeq on real clinical samples, we applied it to nanopore metagenomic sequencing of 41 lower respiratory tracts samples from patients with bacterial lower respiratory infections. Sample characteristics and data generation have been previously reported [20]. We used the original authors’ 1% abundance threshold to report pathogenic microbes, ensuring comparability across methods. We also use the same list of clinically significant pathogens, defined in the Definition subsection of their Methods section, when reporting results to ensure direct comparability. The results of quality control and metagenomic classification are visualized in the Additional files 4, 5. The overall sensitivity and specificity of BugSeq across all target pathogens, as compared to a composite of microbial culture and qPCR, was 100% and 99.6% respectively. WIMP had a sensitivity of 100% and specificity of 98.7%. Using the specificity calculation of Charalampous et al. (described in “Methods” section), the specificity of both tools was 83.3%, concordant with the original manuscript. BugSeq reached better concordance with traditional culture results, as compared with the original “What’s In My Pot” (WIMP) analysis, in 3/41 (7.3%) samples (Additional file 1: Table S2). Specifically, samples S8, S15 and S21 each had *S. pneumoniae* detected by WIMP analysis but not by BugSeq or microbial culture*.* Pathogen-specific qPCR on these samples failed to detect *S. pneumoniae*, confirming these findings [20]. Additionally, BugSeq reached better concordance with qPCR, but not microbial culture, in 1/41 samples (sample S12), where WIMP detected a false-positive *H. influenzae* not detected by BugSeq or qPCR. All other samples were concordant between BugSeq and WIMP. We note that several samples, such as S5 and S28, were incorrect by both BugSeq and WIMP: these samples either had closely related organisms detected (eg. *Klebsiella oxytoca*/*Klebsiella pneumoniae* in S5) or false positives detected by metagenomic sequencing (eg. *S. pneumoniae* in S28).

## Discussion

Here we present BugSeq, an accurate and fast metagenomic classifier for nanopore reads. On simulated data and mock microbial communities, BugSeq was found to outperform MetaMaps, CDKAM and Centrifuge, sometimes by large margins (up to 21%), in terms of precision and recall. On large, 15 GB datasets, BugSeq was also faster than MetaMaps by an order of days, while suffering from a 4-h time trade-off with Centrifuge and CDKAM. BugSeq achieves better classification performance with its reliance on underlying performant tools. Preprocessing relies on fastp, which is optimized for speed by relying on C++ under the hood [11]. Minimap2, which performs BugSeq’s alignment step, is over 30 times faster than most long-read aligners and demonstrated the highest alignment accuracy at the time of its publication [12]. The use of Pathoscope further improves on the read alignments of minimap2, by reassigning reads to their most likely origin using information borrowed from other reads [13]. Finally, by taking the lowest-common ancestor of read assignments, we overcome the classification uncertainty of the previous steps to yield a taxonomic classification that fits with all of the available data.

The results of our study are concordant with existing literature on long-read metagenomic classifiers [9, 10, 23]. We found a lower sensitivity for Centrifuge on the ZymoBIOMICS datasets, which could be attributed to cases in which Centrifuge returns multiple assignments for a single read and collapses these up the taxonomic tree via lowest common ancestor [4]. Similarly, the original MetaMaps paper identified a RAM use of 262 GB and 209 CPU hours for a random sample of a third of the Zymo dataset [8]. In our experience, MetaMaps mapped reads relatively quickly, in accordance with published data on its MinHash-based aligner, but stalled on its “classification” step [24].

In addition to superior performance, part of BugSeq’s innovation lies in its deployment to the cloud for automatic metagenomic analysis from raw reads to report. BugSeq scales with the user’s data, and enables any microbiology laboratory to perform long-read metagenomic classification. BugSeq’s user interface only requires a simple upload of FASTQ files to its website, and returns to the user intuitive HTML files for visualization in their browser. We demonstrate the ease of use of BugSeq by uploading metagenomic data from 41 lower respiratory tract samples. Resulting data, including quality control and metagenomic classification, was packaged into two HTML files (Additional files 4, 5), and showed superior accuracy compared with the original WIMP analysis on the same data. We note, however, limitations in this analysis given the biased nature of culture and qPCR, which will only reveal pathogens of interest.

BugSeq’s main limitation is its execution time and processing requirements, which are greater than Centrifuge and CDKAM. The main reason for the greater processing requirements is that BugSeq performs full read alignments against all of RefSeq, whereas Centrifuge and CDKAM perform substring or k-mer matching against compressed databases. These limitations can partly be overcome by further scaling BugSeq in the cloud. For example, this evaluation was limited to 32 threads for all computations; however, Amazon EC2 now contains instances with up to 448 CPUs. In our experience, minimap2 scales well until at least 64 threads; future work will examine scaling computation to larger EC2 instances for faster analyses.

## Conclusion

BugSeq is a rapid, scalable and accurate metagenomics classifier that outperforms alternatives such as MetaMaps and Centrifuge across a range of performance indicators. BugSeq is deployed to the cloud for easy metagenomic analyses.

## Availability and requirements

- Project name: BugSeq
- Project home page: https://bugseq.com/free
- Operating system(s): Platform independent
- Programming language: Nextflow
- Other requirements: Modern internet browser such as Firefox, Chrome, Safari or Edge
- License: https://docs.bugseq.com/legal/terms-of-use/
- Any restrictions to use by non-academics: Licence required

## Supplementary Information

**Additional file 1.** Supplementary methods, and supplementary tables 1-2.

**Additional file 2**. Krona plot from the BugSeq metagenomic classification of the ZymoBIOMICS mock microbial community with logarithmic organism abundance.

**Additional file 3**. Krona plot from the BugSeq metagenomic classification of the ZymoBIOMICS mock microbial community with even organism abundance.

**Additional file 4**. Aggregated quality control report generated by BugSeq on the nanopore metagenomic sequencing data from Charalampous et al. Forty-one lower-respiratory tract samples are included in the analysis.

**Additional file 5**. Krona plots for BugSeq metagenomic classification of the nanopore metagenomic sequencing data from Charalampous et al. Forty-one lower-respiratory tract samples are included in the analysis.

**Additional file 6**. A screenshot of the graphical user interface of BugSeq. Users may submit data on this screen by clicking “Select Files” or dragging their files into the box, followed by clicking the submit button.

## Data Availability

BugSeq analysis is available at https://bugseq.com/free. Documentation is available at https://docs.bugseq.com. CAMISIM simulated data is available at https://doi.org/10.5281/zenodo.4382659. The ZymoBIOMICS mock community sequencing dataset is available in the SRA repository at accession ERR3152364 and ERR3152366. The bacterial lower respiratory tract sequencing dataset is available in the SRA repository at PRJEB30781.

## Abbreviations

HTML: Hypertext markup language
qPCR: Quantitative polymerase chain reaction
WIMP: What’s In My Pot
