# Supplementary material for: BugSeq: a highly accurate cloud platform for long-read metagenomic analyses
Source: BMC Bioinformatics. 2021 Mar 25;22:160. doi: 10.1186/s12859-021-04089-5 (PMC7993542; doi:10.1186/s12859-021-04089-5)
Supplement: Supplementary file 1 — Additional file 1. Supplementary methods, and supplementary tables 1-2. [file 12859_2021_4089_MOESM1_ESM.docx]

**Supplementary Information**

| **No** | **Taxonomy ID (species)** | **Species** | **NCBI Phylogeny Database** |
| --- | --- | --- | --- |
| 1 | 1280 | *Staphylococcus aureus* | Bacteria; Firmicutes; Bacilli; Bacillales; Staphylococcaceae; Staphylococcus |
| 2 | 1351 | *Enterococcus faecalis* | Bacteria; Firmicutes; Bacilli; Lactobacillales; Enterococcaceae; Enterococcus |
| 3 | 1423 | *Bacillus subtilis* | Bacteria; Firmicutes; Bacilli; Bacillales; Bacillaceae; Bacillus; Bacillus subtilis group |
| 4 | 1613 | *Lactobacillus/Limosilactobacillus fermentum* | Bacteria; Firmicutes; Bacilli; Lactobacillales; Lactobacillaceae; Lactobacillus/Limosilactobacillus |
| 5 | 1639 | *Listeria monocytogenes* | Bacteria; Firmicutes; Bacilli; Bacillales; Listeriaceae; Listeria |
| 6 | 287 | *Pseudomonas aeruginosa* | Bacteria; Proteobacteria; Gammaproteobacteria; Pseudomonadales; Pseudomonadaceae; Pseudomonas; Pseudomonas aeruginosa group |
| 7 | 28901 | *Salmonella enterica* | Bacteria; Proteobacteria; Gammaproteobacteria; Enterobacteriales; Enterobacteriaceae; Salmonella |
| 8 | 4932 | *Saccharomyces cerevisiae* | Eukaryota; Opisthokonta; Fungi; Dikarya; Ascomycota; saccharomyceta; Saccharomycotina; Saccharomycetes; Saccharomycetales; Saccharomycetaceae; Saccharomyces |
| 9 | 5207 | *Cryptococcus neoformans* | Eukaryota; Opisthokonta; Fungi; Dikarya; Basidiomycota; Agaricomycotina; Tremellomycetes; Tremellales; Tremellaceae; Filobasidiella; Filobasidiella/Cryptococcus neoformans species complex |
| 10 | 562 | *Escherichia coli* | Bacteria; Proteobacteria; Gammaproteobacteria; Enterobacteriales; Enterobacteriaceae; Escherichia |

Supplementary Table 1: Organisms present in ZymoBIOMICS mock communities.

|  | **ZymoBIOMICS Microbial Community Standards Dataset** | **Level** | **Mean F-Score** | **F-Score** | **Mean Precision** | **Precision, (%)** | **Mean Recall** | **Recall** |
| --- | --- | --- | --- | --- | --- | --- | --- | --- |
| BugSeq | Even | Kingdom | 0.95 | 0.95 | 99.95 | 100 | 90.53 | 90.69 |
|  |  | Phylum |  | 0.95 |  | 100 |  | 90.61 |
|  |  | Order |  | 0.95 |  | 100 |  | 90.57 |
|  |  | Family |  | 0.95 |  | 100 |  | 90.54 |
|  |  | Genus |  | 0.95 |  | 99.89 |  | 90.43 |
|  |  | Species |  | 0.95 |  | 99.82 |  | 90.36 |
|  | Log | Kingdom | 0.95 | 0.95 | 100 | 100 | 90.90 | 90.94 |
|  |  | Phylum |  | 0.95 |  | 100 |  | 90.90 |
|  |  | Order |  | 0.95 |  | 100 |  | 90.89 |
|  |  | Family |  | 0.95 |  | 100 |  | 90.89 |
|  |  | Genus |  | 0.95 |  | 100 |  | 90.88 |
|  |  | Species |  | 0.95 |  | 100 |  | 90.88 |
|  | Even | Kingdom | 0.92 | 0.98 | 96.72 | 99.95 | 88.05 | 96.12 |
|  |  | Phylum |  | 0.96 |  | 98.82 |  | 93.77 |
|  |  | Order |  | 0.94 |  | 96.93 |  | 90.75 |
|  |  | Family |  | 0.93 |  | 96.35 |  | 89.99 |
|  |  | Genus |  | 0.92 |  | 95.26 |  | 88.53 |
|  |  | Species |  | 0.79 |  | 93.00 |  | 69.17 |
|  | Log | Kingdom | 0.95 | 0.98 | 97.51 | 99.94 | 92.35 | 96.01 |
|  |  | Phylum |  | 0.97 |  | 98.82 |  | 94.38 |
|  |  | Order |  | 0.94 |  | 97.13 |  | 91.92 |
|  |  | Family |  | 0.94 |  | 96.71 |  | 91.46 |
|  |  | Genus |  | 0.94 |  | 96.51 |  | 91.24 |
|  |  | Species |  | 0.92 |  | 95.93 |  | 89.07 |
| Centrifuge –min-hit 22 | Even | Kingdom | 0.92 | 0.96 | 97.72 | 99.97 | 86.36 | 91.90 |
|  |  | Phylum |  | 0.95 |  | 99.25 |  | 91.06 |
|  |  | Order |  | 0.94 |  | 98.13 |  | 89.69 |
|  |  | Family |  | 0.93 |  | 97.65 |  | 89.17 |
|  |  | Genus |  | 0.92 |  | 96.63 |  | 87.80 |
|  |  | Species |  | 0.80 |  | 94.71 |  | 68.53 |
|  | Log | Kingdom | 0.95 | 0.97 | 98.33 | 99.95 | 91.51 | 93.99 |
|  |  | Phylum |  | 0.96 |  | 99.21 |  | 93.12 |
|  |  | Order |  | 0.95 |  | 98.14 |  | 91.37 |
|  |  | Family |  | 0.94 |  | 97.85 |  | 91.04 |
|  |  | Genus |  | 0.94 |  | 97.71 |  | 90.90 |
|  |  | Species |  | 0.93 |  | 97.14 |  | 88.65 |
| MetaMaps (miniSeq+H) | Even | Kingdom | 0.89 | 0.90 | 99.58 | 99.89 | 81.04 | 81.33 |
|  |  | Phylum |  | 0.90 |  | 99.85 |  | 81.21 |
|  |  | Order |  | 0.89 |  | 99.56 |  | 81.05 |
|  |  | Family |  | 0.89 |  | 99.51 |  | 81.01 |
|  |  | Genus |  | 0.89 |  | 99.51 |  | 80.96 |
|  |  | Species |  | 0.89 |  | 99.13 |  | 80.71 |
|  | Log | Kingdom | 0.94 | 0.94 | 99.65 | 99.69 | 88.33 | 88.46 |
|  |  | Phylum |  | 0.94 |  | 99.92 |  | 88.39 |
|  |  | Order |  | 0.94 |  | 99.54 |  | 88.31 |
|  |  | Family |  | 0.94 |  | 99.51 |  | 88.29 |
|  |  | Genus |  | 0.94 |  | 99.80 |  | 88.29 |
|  |  | Species |  | 0.94 |  | 99.46 |  | 88.25 |
| Metamaps (Refseq) | Even | Kingdom | N/A – Out of RAM | | | | | |
|  |  | Phylum |  |  |  |  |  |  |
|  |  | Order |  |  |  |  |  |  |
|  |  | Family |  |  |  |  |  |  |
|  |  | Genus |  |  |  |  |  |  |
|  |  | Species |  |  |  |  |  |  |
|  | Log | Kingdom | 0.94 | 0.94 | 99.81 | 99.98 | 88.72 | 88.88 |
|  |  | Phylum |  | 0.94 |  | 99.84 |  | 88.74 |
|  |  | Order |  | 0.94 |  | 99.77 |  | 88.69 |
|  |  | Family |  | 0.94 |  | 99.76 |  | 88.68 |
|  |  | Genus |  | 0.94 |  | 99.75 |  | 88.67 |
|  |  | Species |  | 0.94 |  | 99.73 |  | 88.66 |
| CDKAM (Standard) | Even | Kingdom | 0.94 | 0.95 | 98.96 | 99.97 | 89.20 | 90.21 |
|  |  | Phylum |  | 0.95 |  | 99.82 |  | 90.08 |
|  |  | Order |  | 0.94 |  | 99.6 |  | 89.88 |
|  |  | Family |  | 0.94 |  | 99.24 |  | 89.54 |
|  |  | Genus |  | 0.94 |  | 98.60 |  | 88.97 |
|  |  | Species |  | 0.91 |  | 96.52 |  | 86.53 |
|  | Log | Kingdom | 0.95 | 0.95 | 99.66 | 99.97 | 89.92 | 90.25 |
|  |  | Phylum |  | 0.95 |  | 99.88 |  | 90.16 |
|  |  | Order |  | 0.95 |  | 99.75 |  | 90.04 |
|  |  | Family |  | 0.95 |  | 99.66 |  | 89.96 |
|  |  | Genus |  | 0.95 |  | 99.62 |  | 89.91 |
|  |  | Species |  | 0.94 |  | 99.06 |  | 89.20 |

**Supplementary Table 2:** Full performance characteristics on ZymBIOMICS mock communities.

| Number of ZymoBIOMICS Log reads | Wall clock time (HH:MM) |
| --- | --- |
| 10^3^ | 00:44 |
| 10^4^ | 00:49 |
| 10^5^ | 01:01 |
| 10^6^ | 02:31 |

**Supplementary Table 3:** BugSeq run time by input size.

| Sample | Sample type | Organism cultured by microbiology | Organism identified by BugSeq | Organism identified from original metagenomic pipeline (WIMP) | qPCR |
| --- | --- | --- | --- | --- | --- |
| S1 | ETA | *E. coli* | *E. coli* | *E. coli* |  |
| S2 | Sputum | *K. pneumoniae* | *K. pneumoniae* | *K. pneumoniae* |  |
| S3 | Sputum | *P. aeruginosa* | *P. aeruginosa* | *P. aeruginosa* |  |
| S4 | Sputum | *S. marcescens* | *S. marcescens* | *S. marcescens* |  |
| S5 | Sputum | *K. oxytoca* | *K. pneumoniae/K. oxytoca* | *K. pneumoniae/K. oxytoca* | *K. pneumoniae* not detected. |
| S6 | Sputum | *S. aureus* | *S. aureus* | *S. aureus* |  |
| S7 | Sputum | *H. influenzae* | *H. influenzae/P. aeruginosa* | *H. influenzae/P. aeruginosa* | *P. aeruginosa* detected. |
| **S8** | **Sputum** | ***M. catarrhalis*** | ***M. catarrhalis*** | ***M. catarrhalis/S. pneumoniae*** | ***S. pneumoniae* not detected.** |
| S9 | Sputum | *P. aeruginosa/E. coli* | *E. coli* | *E. coli* | *P. aeruginosa* not detected. |
| S10 | Sputum | NSG | *H. influenzae/S. pneumoniae* | *H. influenzae/S. pneumoniae* | *H. influenzae* detected.  *S. pneumoniae* detected. |
| S11 | Sputum | NRF | *S. pneumoniae* | *S. pneumoniae* | *S. pneumoniae* detected. |
| **S12** | **Sputum** | **NRF** | ***M. catarrhalis*** | ***H. influenzae/M. catarrhalis*** | ***H. influenzae* not detected. *M. catarrhalis* detected*.*** |
| S13 | Sputum | *S. marcescens* | *S. marcescens* | *S. marcescens* |  |
| S14 | Sputum | *S. aureus* | *S. aureus/M. catarrhalis* | *S. aureus/M. catarrhalis* | *M. Catarrhalis* detected*.* |
| **S15** | **Sputum** | ***S. aureus*** | ***S. aureus*** | ***S. aureus/S. pneumoniae*** | ***S. pneumoniae* not detected.** |
| S16 | Sputum | *S. aureus* | *S. aureus* | *S. aureus* |  |
| S17 | Sputum | NRF | NRF | None |  |
| S18 | Sputum | *H. influenzae* | *H. influenzae* | *H. influenzae* |  |
| S19 | Sputum | NRF | NRF | None |  |
| S20 | Sputum | *H. influenzae* | *H. influenzae* | *H. influenzae* |  |
| **S21** | **Sputum** | **NRF** | ***H. influenzae*** | ***H. influenzae/S. pneumoniae*** | ***H. influenzae* detected.**  ***S. pneumoniae* not detected.** |
| S22 | Sputum | NRF | NRF | None |  |
| S23 | Sputum | *H. influenzae* | *H. influenzae* | *H. influenzae* |  |
| S24 | Sputum | *H. influenzae* | *H. influenzae* | *H. influenzae* |  |
| S25 | Sputum | *H. influenzae* | *H. influenzae* | *H. influenzae* |  |
| S26 | Sputum | *M. catarrhalis* | *M. catarrhalis* | *M. catarrhalis* |  |
| S27 | Sputum | *H. influenzae/S. aureus* | *H. influenzae/S. aureus/S. pyogenes* | *H. influenzae/S. aureus/S. pyogenes* | *S. pyogenes* detected. |
| S28 | Sputum | NRF | *S. pneumoniae* | *S. pneumoniae* | *S. pneumoniae* not detected. |
| S29 | Sputum | *P. aeruginosa* | *P. aeruginosa/S. aureus* | *P. aeruginosa/S. aureus* | *S. aureus* detected. |
| S30 | BAL | *P. aeruginosa* | *P. aeruginosa* | *P. aeruginosa* |  |
| S31 | Sputum | NRF | *H. influenzae* | *H. influenzae* | *H. influenzae* detected. |
| S32 | Sputum | NSG | *E. coli* | *E. coli* | *E. coli* detected. |
| S33 | Sputum | NRF | NRF | None |  |
| S34 | Sputum | NSG | None | None |  |
| S35 | Sputum | *E. coli* | *E. coli* | *E. coli* |  |
| S36 | Sputum | *H. influenzae* | *H. influenzae* | *H. influenzae* |  |
| S37 | Sputum | *P. aeruginosa* | *P. aeruginosa* | *P. aeruginosa* |  |
| S38 | Sputum | *S. aureus/P. aeruginosa* | *S. aureus/P. aeruginosa* | *S. aureus/P. aeruginosa* |  |
| S39 | Sputum | *H. influenzae* | *H. influenzae/M. catarrhalis* | *H. influenzae/M. catarrhalis* | *M. Catarrhalis* detected*.* |
| S40 | ETA | *S. aureus* | *S. aureus* | *S. aureus* |  |
| S41 | Sputum | *H. influenzae/S. aureus* | *H. influenzae/S. aureus* | *H. influenzae/S. aureus* |  |

**Supplementary Table 4:** Performance characteristics on lower respiratory tract specimens. A 1% or greater abundance of any clinically significant microbe (defined by UK Standards for Microbiology Investigations) in a sample was called as present, as performed in the original publication. Differences in microbial identification are bolded. NRF=Normal respiratory flora. NSG=No significant growth.
