# Supplementary material for: BugSeq: a highly accurate cloud platform for long-read metagenomic analyses
Source: BMC Bioinformatics. 2021 Mar 25;22:160. doi: 10.1186/s12859-021-04089-5 (PMC7993542; doi:10.1186/s12859-021-04089-5)
Supplement: Supplementary file 2 — Additional file 2. Krona plot from the BugSeq metagenomic classification of the ZymoBIOMICS mock microbial community with logarithmic organism abundance. [file 12859_2021_4089_MOESM2_ESM.html]

 Javascript must be enabled to view this page.

countunassignedtidrankscoreERR3152366 3422683875271no\_rank62.7330865914002superkingdom64.31760481224phylum61.21760441236class61.217255272274order61.12468family5.52469genus5.51129430species6.01196816no\_rank5.0112662362species5.0172550135621family61.117255058286genus61.1172449136841species\_group61.2172449172212287species61.222381754strain18.011388272strain17.02222941193strain46.01171171009714strain41.5221093787strain34.011111123015strain39.1111193501strain56.0111340851strain8.0441352354strain59.2221400868strain33.5111408274strain14.0221408276strain21.0331411700strain22.7331427342strain16.044441448140strain44.521211457392strain39.51136843species\_group9.011294species9.01136846species\_group5.01147886species5.041196821no\_rank34.941412545800species34.9349291347order62.834925543family62.81547genus19.011354276species\_group19.01791561genus63.017911725562species63.0291483333strain58.222511145no\_rank60.513131245474no\_rank64.2183334serotype16.0111330457strain16.011340186strain20.011469008strain59.044941323strain50.0111038927serotype56.0111050617strain58.0222048781strain23.0112603836serotype24.023232605619no\_rank61.7222773706no\_rank37.51570genus64.01573species64.01172407subspecies64.01694590genus62.81694728901species62.8168720159201subspecies62.944600no\_rank40.02611no\_rank41.5111124936strain36.0111299044strain47.0129482no\_rank28.0111029983strain28.0157045no\_rank20.011224729strain20.0157743no\_rank52.0111173939strain52.02258712no\_rank47.01190370no\_rank63.01421142190371no\_rank63.211108619no\_rank38.0405119912no\_rank63.811321314strain43.011904139strain83.03333938142strain65.122149391no\_rank59.065149539no\_rank48.0111412522strain63.011192953no\_rank33.011224727no\_rank47.011340189no\_rank28.01913076no\_rank10.0111242101strain10.0228211class10.01204457order9.0141297family9.0113687genus9.01196159no\_rank9.0111517551species9.01204458order11.0176892family11.0175genus11.01169666species11.0228216class49.0280840order49.0280864family49.01283genus35.01285species35.0111392005strain35.01219181genus63.012645081no\_rank63.0111658672species63.011783270no\_rank32.0168336no\_rank32.01976phylum32.01117743class32.01200644order32.012762318family32.01501783genus32.011237258species32.031312101783272no\_rank64.431312021239phylum64.4313120124891061class64.431292631491385order64.4290964family53.521279genus53.5221280species53.5381911186817family63.838190151386genus63.85286661species\_group41.6211396species38.511526969strain61.0111428species22.02185979no\_rank17.5111837130species15.0112009331species20.0381575653685species\_group63.838145381151423species63.81996241subspecies29.888655816strain34.81111703612strain26.244135461subspecies49.833483913subspecies32.0111204342strain72.0111220533strain11.0221415167strain9.061653388species\_subgroup50.54472360species51.511260554species28.011938374species\_subgroup42.01492670species42.0111458206strain42.011111963032species19.92186818family15.011372genus7.012662419no\_rank7.0112058136species7.01651660genus23.012642018no\_rank23.0112320858species23.03090919186820family64.53090919231637genus64.52481638species41.61616202752subspecies49.1309084729405311639species64.57171393118strain50.099393119strain20.411393127strain11.099863767strain33.057095709879088strain48.1120120879089strain47.822879090strain17.011882020strain8.04141882094strain37.0170170882095strain46.316581658930781strain51.499930782strain23.94545932919strain44.66806801027396strain49.252521126011strain40.51431431299895strain43.025251437838strain32.751559515591457188strain58.3111906951serotype77.08997757342065118serotype54.918331833265669strain46.63030568819strain44.7134413441196159strain44.351089510891196160strain58.9144114411196161strain43.7426842681196162strain47.613198131981196171strain52.7179017901196172strain44.2418241821196173strain48.22692691196174strain48.02362361196175strain48.7456345631230340strain48.03422291966serotype36.4771196163strain28.925251196164strain40.9551640species14.81671642species19.433272626strain22.766702455strain9.0331643species17.3111006155species5.016901186826order67.441300family23.0321301genus27.311303species26.011655813strain26.0111357genus10.017633958family59.411578genus44.01183683species44.01742742598genus59.71741741613species59.712759736genus19.0111597species19.0150881852family68.515081350genus68.5150815071351species68.511565651strain20.01186828family58.0112747genus58.01186801class40.01186802order40.0131984family40.0179598genus40.011356322species40.08201174phylum47.471760class50.7285006order68.52145357family68.5257495genus68.5221274species68.5585009order43.6431957family51.541912216genus51.5441747species51.5185015family12.011839genus12.012615069no\_rank12.0112712223species12.0184995class24.0184996order24.0184997family24.0142255genus24.0149319species24.011266117strain24.032157superkingdom30.7328890phylum30.732290931no\_rank30.73183963class30.731644055order30.731644056family30.732251genus30.733255616species30.7264082759superkingdom62.52640533154no\_rank62.5263664751kingdom62.526366451864subkingdom62.5263394890phylum62.526339716545no\_rank62.526338147537subphylum62.5263384891class62.5263384892order62.52633814893family62.5263354930genus62.5263024932species62.62630226302559292strain62.6333327291species46.014948genus15.01148254species15.0133170genus15.01145286species15.01147538subphylum7.011716546no\_rank7.0275204phylum57.3275302subphylum57.327155616class57.3275234order57.3271884633family57.3235206genus59.1231897064species\_group59.1235207species59.117540410variety55.677214684strain52.055283643strain58.86178876variety68.866235443strain68.84490731genus46.8441734106species46.83933208kingdom39.0396072no\_rank39.03933213no\_rank39.03933511no\_rank39.0397711phylum39.03989593subphylum39.0397742no\_rank39.0397776no\_rank39.039117570no\_rank39.039117571no\_rank39.0398287superclass39.0391338369no\_rank39.03932523no\_rank39.03932524no\_rank39.03940674class39.03932525no\_rank39.0399347no\_rank39.0391437010no\_rank39.039314146superorder39.0399443order39.039376913suborder39.039314293infraorder39.0399526parvorder39.039314295superfamily39.0399604family39.039207598subfamily39.0399605genus39.039399606species39.032698737no\_rank6.0333630no\_rank6.035794phylum6.031280412class6.035796subclass6.0375739order6.03423054suborder6.035809family6.035810genus6.035811species6.033508771strain6.0862787854no\_rank50.58628384no\_rank50.58681077no\_rank50.5868632630species50.5
