# Supplementary material for: BugSeq: a highly accurate cloud platform for long-read metagenomic analyses
Source: BMC Bioinformatics. 2021 Mar 25;22:160. doi: 10.1186/s12859-021-04089-5 (PMC7993542; doi:10.1186/s12859-021-04089-5)
Supplement: Supplementary file 3 — Additional file 3. Krona plot from the BugSeq metagenomic classification of the ZymoBIOMICS mock microbial community with even organism abundance. [file 12859_2021_4089_MOESM3_ESM.html]

 Javascript must be enabled to view this page.

countunassignedtidrankscoreERR3152364 3263590970981no\_rank64.3301743424912superkingdom66.26073981224phylum65.26073951811236class65.218588672274order64.1185886135621family64.118588614286genus64.1185865136841species\_group64.1185865185547287species64.11919208964strain55.488388272strain47.911557722strain26.022910265strain20.511941193strain15.02192191009714strain48.8111093787strain18.0221123015strain69.524241193501strain43.5771279007strain42.0771279008strain48.114141356855strain52.6221400868strain21.5111408273strain43.0111408275strain37.0111415629strain7.0881427342strain42.22136843species\_group13.022294species13.05196821no\_rank59.4111978467species68.0442545800species57.2421327191347order65.8421323317543family65.89544genus18.32235703species33.031344959species\_group11.322546species11.51157706species11.0111563222species25.032644389no\_rank13.3112742622species7.0112742635species26.0112742653species7.04547genus33.04354276species\_group33.02158836species27.0221812934subspecies27.011208224species45.0111812935species33.021063511561genus66.2210603201280562species66.23337762strain52.06195343783333strain65.111316385no\_rank61.033316407no\_rank31.314451445511145no\_rank65.011679895no\_rank13.013071307879462no\_rank65.4111245474no\_rank52.0555083334serotype46.2551328859strain60.611244319serotype16.06244320serotype67.822701177strain78.0441048689strain62.855316401strain34.444331112strain43.22020340186strain54.411362663strain15.01211376725serotype51.011585395no\_rank14.011409438strain40.044413997strain67.25757481805strain55.5127127498388strain62.41919536056strain58.144566546strain56.211577675strain38.022585035strain43.577585055strain53.33737595495strain48.266696406strain54.3101101745156strain61.36861906serotype41.866216592strain41.844866789strain54.822913091strain62.599930406strain37.211941322strain21.0544544941323strain63.667431038927serotype48.010101048254strain46.0661133852strain45.8221133853strain47.5661134782strain46.7771045010serogroup55.6115711571050617strain63.630301055538serogroup47.4101078034serotype31.710101248915strain31.7441200752strain61.0111329907strain15.0111335916strain61.0881358422strain52.9111382700strain63.0111392858strain48.0221412834strain36.0221435461strain49.5111441627strain19.0221446701strain54.5221446707serotype58.538381446746serotype60.161603259serotype45.566331111strain45.5112027293serotype47.010102048777strain38.7222048780strain60.525252048781strain43.442233553serogroup47.8441055541no\_rank47.810102603836serotype62.25435432605619no\_rank62.81071072773705no\_rank59.941412773706no\_rank59.0662773707no\_rank43.21212564species30.655208962species37.0331499973species8.712608889no\_rank36.0112044467species36.09570genus36.977573species28.911244366species75.0111134687species55.021022764590genus65.4210100203528901species65.420804511640259201subspecies65.61594no\_rank8.011550538strain8.0173152595no\_rank44.52121596155strain41.03131596no\_rank37.9160133600no\_rank44.22525935705strain48.8221064551strain48.017604no\_rank56.5771081093strain50.410101225522strain60.791605no\_rank37.9881029979strain38.110381611no\_rank46.517171124936strain54.1551271864strain44.4838328142no\_rank46.99928144no\_rank37.9434328147no\_rank39.2202028150no\_rank39.52229474no\_rank66.54329482no\_rank41.243431029983strain41.2747448409no\_rank45.2131254388no\_rank31.511295319strain37.031257045no\_rank45.31515224729strain44.514141016998strain46.625757046no\_rank48.6257257476213strain48.6161657741no\_rank39.4121157743no\_rank39.0111173939strain15.0635758095no\_rank40.4111072590strain20.0551406860strain39.619010958096no\_rank45.2111173456strain5.0221182172strain23.510101182174strain43.628281182177strain42.9111182178strain53.0441182180strain45.218181183391strain40.0881208622strain54.2221208629strain42.5771208630strain39.11158101no\_rank24.019216658712no\_rank46.177984211strain52.1441399029strain22.0111454583strain13.0661454585strain41.8221454586strain58.0111454589strain41.0221454590strain11.0221454593strain7.5111454596strain26.07682689no\_rank52.7111124958strain71.019819590105no\_rank46.833702982strain19.710710590370no\_rank41.422220341strain59.0403384026290371no\_rank65.96199287strain50.255588858no\_rank51.899568708strain41.311996633strain75.0111008297strain75.0111029978strain42.0111454636strain74.0111454640strain28.0111454645strain18.053531454647strain44.3221620419no\_rank46.51213898360no\_rank46.266439851strain49.57676687860strain48.811909945strain34.0319167108619no\_rank45.877796730strain50.433796731strain38.722796732strain55.588858305strain61.01313858306strain51.355858307strain38.21313930778strain47.82525930779strain40.577997339strain43.1881454614strain51.624241454618strain40.0331454619strain32.7881454620strain41.810101454625strain42.016161454627strain40.1116115981no\_rank35.5221454601strain37.0331454608strain44.71313117541no\_rank45.34678127711119912no\_rank64.8285285321314strain48.4287287904139strain48.61849818498938142strain65.111134047no\_rank66.088143221no\_rank34.0101101149385no\_rank45.81149386no\_rank5.011941190strain5.044149388no\_rank23.855149390no\_rank37.010378149391no\_rank45.12525930771strain45.4716594149539no\_rank51.611550537strain74.055886715strain59.088998822strain67.5111243621strain65.0221244111strain55.533331244122strain53.2111412451strain36.028281412455strain53.1551412490strain53.4221412492strain54.5661412495strain47.2771412499strain64.3111412507strain71.0111412515strain75.010101412525strain45.5441412529strain57.8111412571strain77.0661412586strain43.73181798no\_rank57.033938141strain57.05189201no\_rank38.6551271863strain38.68787192953no\_rank47.2116192954no\_rank35.955984237strain41.652192955no\_rank62.2331242102strain66.72323211968no\_rank43.83838224727no\_rank40.51919260367no\_rank31.57260368no\_rank33.4771240708strain33.4100100260678no\_rank43.78651286782no\_rank43.235351194159strain48.52525286783no\_rank44.212488340188no\_rank42.536361410916strain49.41717340189no\_rank41.52399583no\_rank77.0221240694strain77.01436295no\_rank25.0111124962strain25.099440524no\_rank48.42727483687no\_rank42.918851486994no\_rank43.21371371242097strain42.611570935no\_rank38.088593905no\_rank45.17913070no\_rank46.0771242094strain46.024913074no\_rank39.42424941187strain39.417913085no\_rank48.517171243595strain48.52915158no\_rank47.5221242108strain47.54040932733no\_rank46.824241077085no\_rank38.936361129117no\_rank45.3111160769no\_rank68.011242079no\_rank70.0111242088strain70.011242081no\_rank69.0111242096strain69.0121242084no\_rank43.312121242106strain43.321242085no\_rank8.0221242107strain8.0201243577no\_rank41.820201243578strain41.81341243585no\_rank39.5991243586strain47.4121243590no\_rank41.012121243591strain41.051243599no\_rank49.2551243600strain49.21851851962641no\_rank45.7111967643no\_rank75.0112021403no\_rank61.0222024273no\_rank56.574742583588no\_rank48.29359202subspecies36.233297361no\_rank32.321243601no\_rank59.0221243602strain59.0112577863no\_rank13.0459203subspecies28.831243605no\_rank16.3331243606strain16.3111243607no\_rank66.04459204subspecies15.5359205subspecies43.03358100no\_rank43.0254736species37.5141527no\_rank22.0111382510strain22.0111197719strain53.0612614656no\_rank51.1552500543species29.855552664291species52.9112686305species61.01204620genus45.13535621species38.744622species15.07471623species49.12424718serotype69.0221086030strain69.0112049486strain61.032629414no\_rank68.0331813821species68.01158483genus19.011158822species19.01160674genus40.011575species40.021903412family29.52635genus29.52267780species29.511903414family11.01583genus11.011584species11.01135623order47.01641family47.01662genus47.011190893species47.0328216class33.3280840order43.0275682family43.02229580genus43.01206351order14.01481family14.01482genus14.011485species14.011783270no\_rank26.0168336no\_rank26.01976phylum26.01117743class26.01200644order26.0149546family26.011237genus26.024075441783272no\_rank66.5240753841239phylum66.52407533121391061class66.514919289661385order67.339303390964family68.5393033981279genus68.53924623913261280species68.5113046546170subspecies56.53393062strain82.055158878strain53.611158879strain80.033282459strain31.711359786strain51.011359787strain32.011418127strain38.0118118426430strain62.122663951strain30.5219219681288strain60.722889933strain36.0661006543strain32.2331074252strain12.71371371074919strain55.2331123523strain23.321196216strain65.0221193576no\_rank65.031311241616strain63.21111111343064strain50.916161381115strain47.944703339strain48.0221458279strain12.5991283species60.0463463283734species56.711985002species15.06360633186817family66.36360592621386genus66.3733886661species\_group55.824111396species47.511222523strain49.011361100strain63.011405535strain77.055526969strain30.833526973strain25.011526992strain69.0111126681strain66.0331428species47.7662026186species61.7222026187species78.5205185979no\_rank26.2551127744species17.4111574141species58.0661837130species38.0222009331species13.0112762329species14.063471592653685species\_group66.36345416335051423species66.37786029subspecies46.482896241subspecies27.1554554655816strain27.1239239703612strain26.035351052585strain34.110590135461subspecies47.188535024strain36.622535025strain51.511535026strain16.0111052588strain11.0331404258strain48.04848483913subspecies43.021211204342strain51.511111220533strain32.016161415167strain32.6321452species54.0111239783strain57.06672361species20.322227866species12.04110653388species\_subgroup46.36672360species55.52525260554species40.63071938374species\_subgroup41.8111390species22.02222492670species43.5111441095species9.0111664069species65.09879871963032species29.312675231genus9.011129985species9.0461865186820family67.746186561637genus67.7331638species50.74618514504261639species67.722393118strain23.533393119strain13.711393130strain12.011863767strain50.0389389879088strain49.333879089strain39.011879090strain9.022882094strain24.51919882095strain40.115061506930781strain60.51919930782strain59.722932919strain44.5551027396strain61.42862861126011strain56.21151151299895strain48.14544541457188strain52.9860739302065118serotype54.4755755265669strain51.011568819strain5.03713711196159strain50.83433431196160strain50.53693691196161strain50.23763761196162strain50.95885881196171strain52.15925921196172strain51.75945941196173strain51.3441196174strain24.2221196175strain55.06826821230340strain52.31022291966serotype57.8441196163strain40.2441196164strain65.2111640species70.0441642species36.21186822family7.0185151no\_rank7.0155079genus7.012642329no\_rank7.0111450761species7.091439225186826order65.341300family32.5431301genus32.51671232species\_group37.0111328species37.05109645433958family64.0321253genus20.0111255species5.0431578genus22.8111584species8.05108822742598genus64.0111598species49.05108775107791613species64.01111334390strain26.81616767453strain26.471711381124strain29.81135787species17.03397478species25.7412759736genus51.0331597species61.322767842genus17.5221590species17.512767879genus71.01183526species71.022767881genus18.0221625species18.052767886genus33.0551580species33.022767887genus39.5111622species44.011624species35.011362948strain35.012767890genus62.0111296540species62.022767892genus35.022240427species35.022767893genus19.511581species29.011511437strain29.0111588species10.0281850family34.511243genus17.011244species17.01115778subspecies17.0111165892strain17.0146255genus52.0112506420species52.040339481852family66.9403391131350genus66.94033674029011351species66.955474186strain26.833565651strain14.733699186strain17.338381157365strain40.21311311201292strain35.42152151206105strain39.8331261557strain11.068681287066strain40.0991352species46.7222005703species16.512737genus46.012648499no\_rank46.0112714947species46.0133969genus54.01133970species54.0151668genus26.01151669species26.01186827family60.0111375genus60.02186828family22.5129393genus9.01129394species9.0111470540genus36.01186801class37.01186802order37.0168298family37.01129001genus37.0186170species37.011643648strain37.06201174phylum46.761760class46.7285004order22.5231953family22.52196081genus22.5278259species22.5221150468strain22.5485009order58.8431957family58.841912216genus58.8441747species58.812157superkingdom39.0128890phylum39.012290931no\_rank39.01183963class39.011644055order39.011644056family39.012251genus39.011255616species39.014876212759superkingdom64.014875633154no\_rank64.01487034751kingdom64.014870330451864subkingdom64.0795364890phylum65.4795361716545no\_rank65.479533147537subphylum65.4795334891class65.47953314892order65.47953114893family65.4795234930genus65.4793774932species65.47937779377559292strain65.414414427291species46.9221080349species17.0214948genus30.01148254species15.02133170genus26.0133169species33.011284811strain33.01278028genus63.0127289species63.0111071378strain63.01374468genus34.01600669no\_rank34.0115478species34.011196389genus71.01142260species71.0134353family7.011232588genus7.0112606893species7.02147538subphylum46.521716546no\_rank46.51147545class52.01451871subclass52.015042order52.011131492family52.0115052genus52.0691375204phylum62.3691365302subphylum62.3691361155616class62.3691355234order62.3691352201884633family62.3658305206genus62.715104669species16.315151295533strain16.3421884637species\_group47.64237769species47.64242367775strain47.6657731897064species\_group62.7657731365207species62.740052648540410variety62.469126912214684strain63.52665526655283643strain61.925585178876variety63.42558525585235443strain63.43085490731genus55.0308530851734106species55.01452284subphylum51.011538075class51.01162474order51.01742845family51.0155193genus51.01176775species51.05333208kingdom55.7536072no\_rank55.75333213no\_rank55.75333511no\_rank55.7537711phylum55.75389593subphylum55.7537742no\_rank55.7537776no\_rank55.753117570no\_rank55.753117571no\_rank55.7538287superclass55.7531338369no\_rank55.75332523no\_rank55.75332524no\_rank55.75340674class55.75332525no\_rank55.7539347no\_rank55.7531437010no\_rank55.753314146superorder55.7539443order55.753376913suborder55.753314293infraorder55.7539526parvorder55.753314295superfamily55.7539604family55.753207598subfamily55.7539605genus55.753539606species55.752698737no\_rank7.0533630no\_rank7.055794phylum7.051280412class7.055796subclass7.0575739order7.05423054suborder7.015799family6.015800genus6.0115804species6.0415809family7.235810genus7.035811species7.033508771strain7.0110239superkingdom63.012731341no\_rank63.012731360no\_rank63.012731618phylum63.012731619class63.0128883order63.0110699family63.01196894no\_rank63.0111458852species63.02942787854no\_rank51.829428384no\_rank51.829481077no\_rank51.829429432630species51.8
