# Supplementary figures and images for: BugSeq: a highly accurate cloud platform for long-read metagenomic analyses
Source: BMC Bioinformatics. 2021 Mar 25;22:160. doi: 10.1186/s12859-021-04089-5 (PMC7993542; doi:10.1186/s12859-021-04089-5)

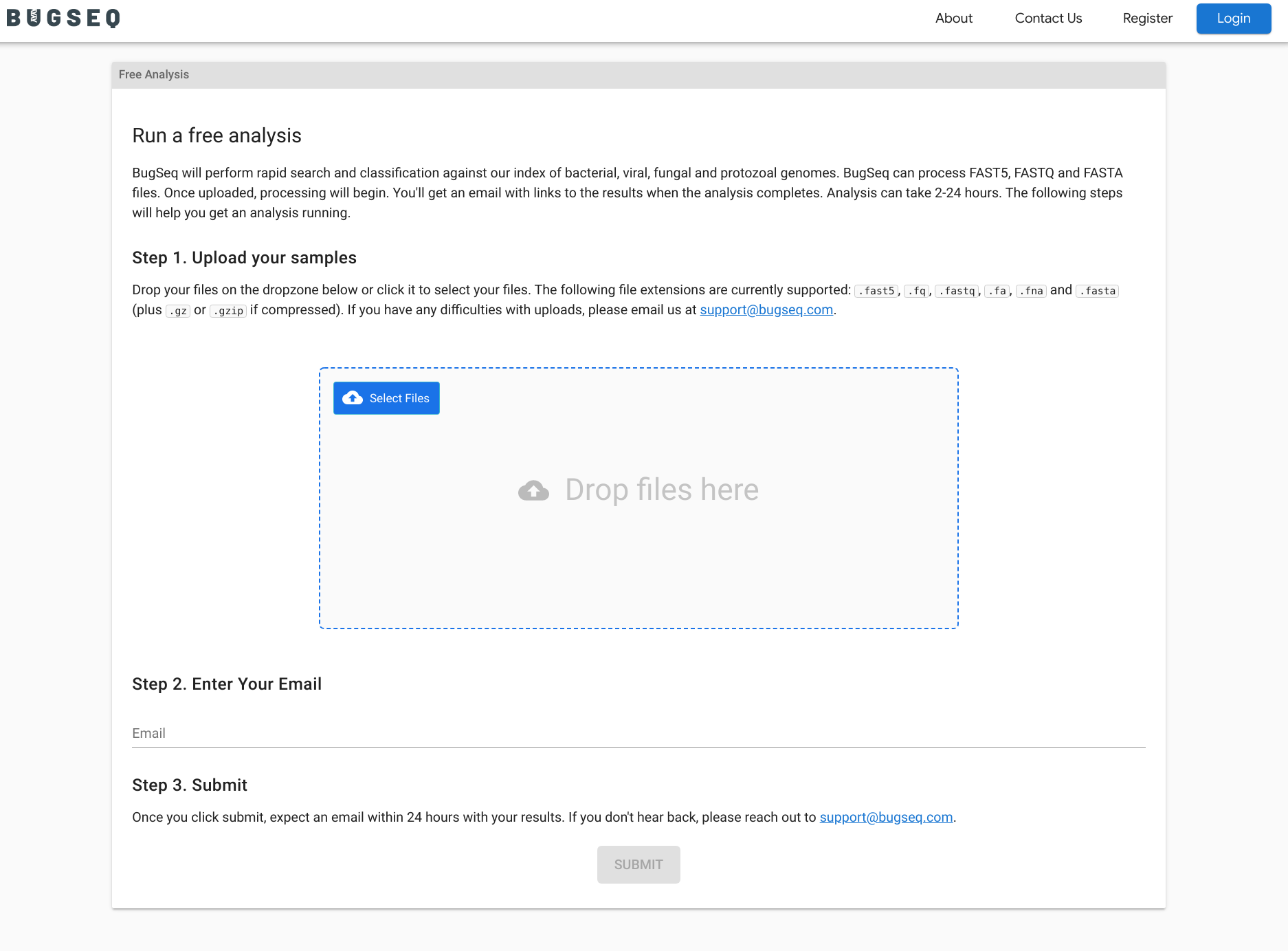

Supplement: Supplementary file 6 — Additional file 6. A screenshot of the graphical user interface of BugSeq. Users may submit data on this screen by clicking “Select Files” or dragging their files into the box, followed by clicking the submit button. [file 12859_2021_4089_MOESM6_ESM.png]
